# Relationship between asporin and extracellular matrix behavior: A literature review

**DOI:** 10.1097/MD.0000000000032490

**Published:** 2022-12-23

**Authors:** Rui Fan, Xiaoyan Yan, Wei Zhang

**Affiliations:** a First School of Clinical Medicine, Shandong University of Traditional Chinese Medicine, Shandong, China; b Department of Geriatrics, Shandong University of Traditional Chinese Medicine Affiliated Hospital, Shandong, China; c Department of Respiratory and Critical Care Medicine, Shandong University of Traditional Chinese Medicine Affiliated Hospital, Shandong, China.

**Keywords:** ASPN, asporin, collagen behavior, epithelial–mesenchymal transition

## Abstract

Asporin (ASPN), as a member of the small leucine-rich repeat proteoglycan family, is a type of protein that is found in the extracellular matrix. Collagen deposition or transformation is involved in a variety of pathological processes. ASPN is identified in cancerous tissue, pathological cardiac tissue, articular cartilage, keloid, and fibrotic lung tissue, and it has a role in the development of cancer, cardiovascular, bone and joint, keloid, and pulmonary fibrosis by interfering with collagen metabolism. This review article summarizes the data on ASPN expressions in mouse and human and highlights that overexpress of ASPN might play a role in a variety of diseases. Although our knowledge of ASPN is currently limited, these instances may help us better understand how it interacts with diseases.

## 1. Introduction

Asporin (ASPN) was the first member of the small leucine-rich proteoglycan (SLRPs) family to be discovered, and it has been linked to collagen fibrillogenesis, signal transduction, and tumor growth.^[1]^ SLRPs play an important role in extracellular matrix (ECM) homeostasis and collagen behavior by regulating collagen fibrillation and binding to transforming growth factor-β (TGF-β) and other ligands and receptors in a complex manner. The SLRPs family consists of 18 members which are classified into 5 classes (I–V) based on the structural properties of their core proteins. ASPN was identified in 2001 as a member of the class I SLRPs by Lorenzo et al,^[2]^ Henry et al,^[3]^ and Yamada et al,^[4]^ which was identified as “periodontal ligament-associated protein 1” because it was specifically expressed in the periodontal ligament.

The Asporin protein has 380 amino acid residues and its amino sequence is very similar to that of the decorin (54%) and biglycan (60%) proteins, all of which belong to the class I SLRPs family.^[5]^ Although most SLRPs proteins are proteoglycans, ASPN cannot be considered a proteoglycan in the strictest sense because it lacks the serine/glycine dipeptide sequence for O-linked glycosaminoglycan binding compared to decorin and biglycan. The main components of ECM are glycoproteins, collagens, and proteoglycans, which form the “core matrix-some” in humans, encompassing nearly 300 proteins. Recent research has concentrated on epithelial–mesenchymal transition (EMT), which is one of the mechanisms underlying the etiology and molecular mechanism of many diseases.^[6–8]^ EMT is a reversible series of molecular events in which epithelial cells acquire a mesenchymal phenotype and invade surrounding tissues.^[9,10]^ Signaling pathways which are mediated by TGF-β and bone morphogenetic protein, Wnt/β-catenin, Notch, Hedgehog, receptor tyrosine kinases, and others induce transcription program switching in EMT.^[10,11]^ To exemplify, EMT mediated pulmonary fibrosis (PF),^[12]^ cardiac repair postmyocardial infarction,^[13]^ osteoarthritis (OA),^[1]^ rheumatoid arthritis,^[14]^ ovarian tumor growth,^[15]^ keloid disorders,^[16]^ and beyond. ASPN was discovered in the ECM of cartilage that surrounds skeletal tissue, but the relationship between ASPN and ECM, however, is not yet clear. Researchers have gradually discovered a closer relationship between ASPN and ECM behavior as clinical and basic research has progressed.^[17]^

## 2. The molecular architecture of ASPN

Small leucine-rich proteoglycans (SLRPs) are a type of ECM that belongs to the protein superfamily leucine-rich repeat (LRR). The LRR is a protein folding motif composed of 20 to 30 amino acids with leucines in conserved positions.^[3]^ ASPN is a member of the LRR protein family, which is associated to cartilage matrix. The name ASPN refers to the protein’s unique aspartate-rich N terminus and its general similarity to decorin.^[2]^ Members of the SLRPs subfamily have core proteins that are similar in size (approximately 40 kDa) and are dominated by a central domain composed of 6 to 10 tandemly repeated LRRs. The other identified class I members, decorin, and biglycan,^[18]^ are sensitively linked to SLRPs based on amino acid sequences. The amino acid sequence and size of the ASPN protein are strikingly similar to those of the decorin and biglycan core proteins. These 3 SLRPs members have some structural similarities and differences, such as different core protein molecular weights (ASPN: 42 kDa; decorin: 36 kDa; biglycan: 38 kDa).^[19,20]^

**Figure 1.**
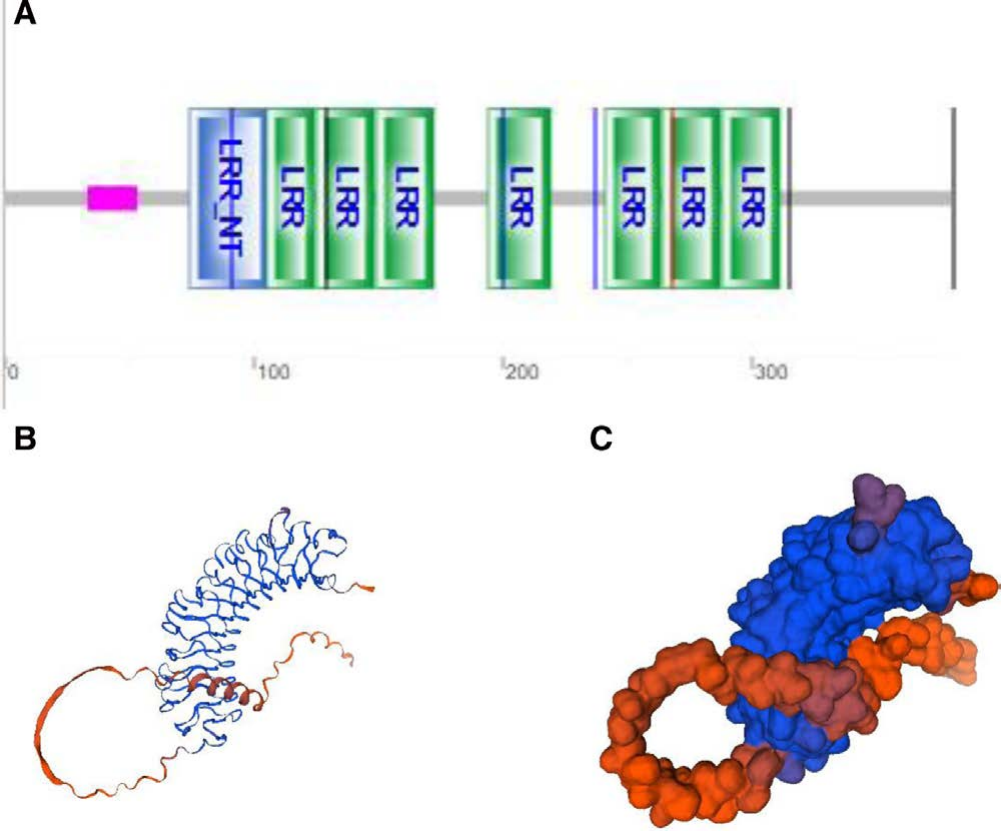
Domains within Homo sapiens protein ASPN_HUMAN (Q9BXN1). Note: (A) Schematic representation *of* Asporin; (B, C) AlphaFold Model of Asporin (AF-Q9BXN1-F1), model confidence: dark blue: very high (pLDDT > 90); light blue: confident (90 > pLDDT > 70); yellow: low (70 > pLDDT > 50); orange: very low (pLDDT < 50), AlphaFold produces a per-residue confidence score (pLDDT) between 0 and 100. Some regions below 50 pLDDT may be unstructured in isolation.

ASPN protein is encoded by ASPN gene, which is located on chromosome 9q22-9q21.3. It’s part of an LRR gene cluster that includes the genes ECM2, OMD, and OGN.^[3]^ Exon 2 of the ASPN gene contains a triplet repeat that codes for a polymorphic stretch of aspartic acid residues in the N-terminal region of the protein. This repeat polymorphism (D-repeat) has 10 alleles encoding 10–19 residues, with the D13 allele being the most common. To date, several studies demonstrated that ASPN D-repeat polymorphism is not associated with increased knee OA risk,^[20]^ while other studies show an obvious association between the D repeat polymorphism of ASPN and dysplasia of the hip (DDH).^[21]^ Asporin *molecular* structures are shown in Figure 1^[22]^ and Table 1.^[23]^

**Table 1:** Confidently predicted domains, repeats, motifs, and features.

Name	Start	End	E-value
Low complexity	33	53	N/A
LRRNT	74	106	0.00000103
LRR	105	124	95
LRR	125	148	0.307
LRR	149	172	5.41
LRR	194	219	92.4
LRR	241	263	276
LRR	264	287	30
LRR	288	311	11.9

LRR = leucine-rich repeat.

## 3. Construction of protein–protein interaction networks

**Figure 2.**
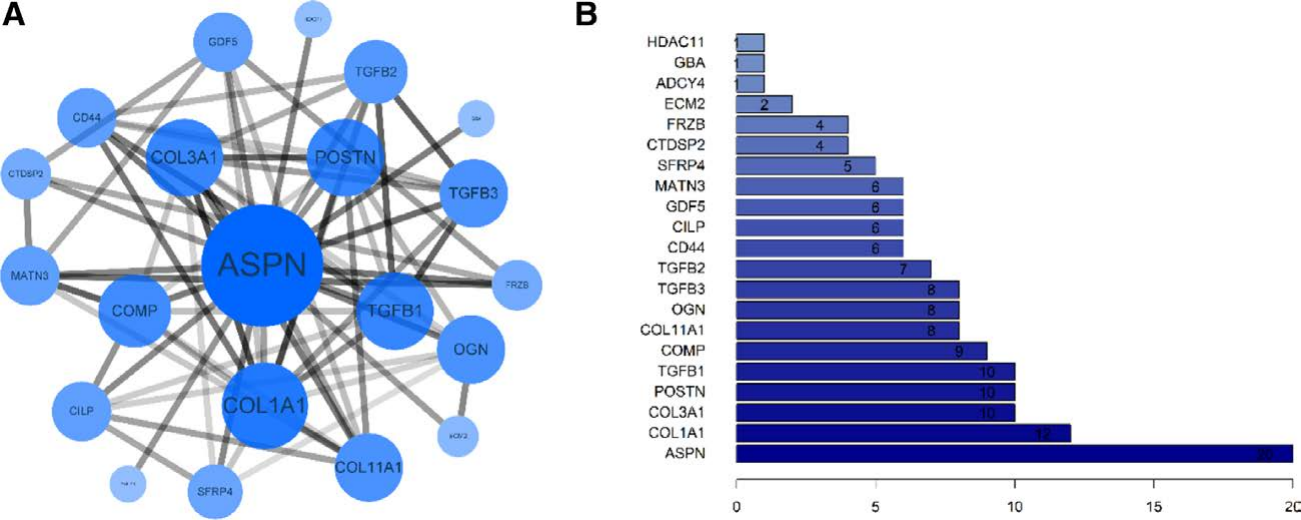
ASPN and its related proteins. Note: (A) PPI network diagram of ASPN; (B) Degree of ASPN and its related genes.

We analyzed protein–protein interaction (PPI) of ASPN using the Search Tool for the Retrieval of Interacting Genes (STRING),^[24]^ a highly cited human PPI database. Then, the Interactions were imported and visualized in Cytoscape v3.9.1 (http://www.cytoscape.org). ASPN protein and its associated proteins are shown in Figure 2. The STRING database provided functional associations for proteins, which were sorted by a confidence score. Data only for “Homo sapiens” with a confidence score ≥ 0.4 (Medium confidence) was used for PPI construction, containing 21 nodes and 72 edges. The network diagram indicated that ASPN had the highest *degree* and was, therefore, the core target gene. Also, a series of high Degree of ASPN-related genes COL1A1, COL3A1, POSTN, TGFB1, COMP, COL11A1, OGN, TGFB3, TGFB2, and GDF5 was identified, and their Degrees were 12, 10, 10, 10, 9, 8, 8, 8, 7, and 6, respectively. Collagen deposition and EMT are associated to these ASPN-related genes, such as COL1A1,^[25]^ TGFB1,^[26]^ COL3A1,^[27]^ COMP,^[28]^ and COL11A1,^[28]^ which indicate ASPN plays a key role in the ECM deposition and EMT process.

## 4. The research hotspots of ASPN

Since its discovery in 2001, researchers have focused on ASPN’s role in bone and joint diseases such as OA, intervertebral disc degeneration (DD), and periodontal ligament mineralization^[1,29–31]^ ASPN expression has recently been found to be dysregulated in tumor tissues and to be positively or negatively associated with tumor proliferation, migration, invasion, and patient prognosis via regulation of numerous signaling pathways, including TGF-β, EGFR, and CD44.^[5]^ ASPN has a wide range of effects on a variety of diseases, for example, it can improve the myocardial remodeling process of dilated cardiomyopathy (DCM) ^[17]^ and is a promising therapeutic target for heart failure (HF),^[32,33]^ inhibit the mechanical communication between fibroblasts mediated by collagen matrix to improve the prognosis of keloid,^[34,35]^ and as one of the characteristic genes of idiopathic PF, it affects the process of PF through TGF-β pathway.^[36]^ The biological law of ASPN has gradually been revealed as research on it has progressed. The primary goal of this review is to summarize ASPN’s role in various systems as well as the relationship between ASPN and ECM behavior.

## 5. Relationship between ASPN and ECM behavior in tumor growth, invasion, and evolution

According to a population-based analysis,^[37]^ cancer is the leading cause of death and a major impediment to increasing life expectancy in every country on the planet in 2020. Lung cancer remained the leading cause of cancer death with an estimated 1.8 million deaths (18%), followed by colorectal cancer (9.4%), liver cancer (8.3%), gastric cancer (7.7%), and female breast cancer (6.9%).^[38]^ Massive studies suggest that ASPN plays an important role in the pathogenesis of various cancers acting as an oncogene, with numerous studies linking ASPN to gastric cancer,^[39,40]^ colon cancer,^[41]^ pancreatic cancer,^[42]^ prostate cancer^[43]^ via EMT process.

Previous studies have shown that exogenous ASPN activated NF-B/p65 and enhanced the EMT process in pancreatic cancer cells through activating the CD44/AKT/ERK/NF-κB pathway in paracrine and autocrine ways.^[42]^ As a result, ASPN may be a prognostic marker for pancreatic cancer, and targeting the tumor microenvironment represents a promising therapeutic strategy.^[42]^ Li et al^[41]^ have confirmed that ASPN expression levels in colon cancer tissues are higher than in normal tissues, and ASPN interacts with Smads protein, promotes Smads protein translocation into the nucleus, and upregulates the expression of genes associated with EMT. Furthermore, ASPN was discovered in the reactive stroma as a stroma-expressed biomarker that was associated with the progression of prostate cancer.^[43]^ Besides that, in a mouse model of prostate cancer, inhibition of p53-induced ASPN expression in the tumor stroma was linked to the expression of neuroendocrine markers.^[43]^ Ding et al^[40]^ discovered that ASPN is overexpressed in gastric cancer tissues compared to noncancerous tissues, and suppressing ASPN inhibits the proliferation and migration of immortalized tumor epithelial cells by influencing the EGFR signaling pathway, which further illustrated the relationship of ASPN to collagen behavior in the realm of cancer. They also reveal that ASPN downregulation has been shown to block the antiapoptotic molecule Bcl-2, increase the expression of the apoptotic molecule Bad, and decrease the expression of migration-related proteins CD44 and matrix metalloproteinases.^[40]^ In addition, ASPN promotes the invasion of adjacent cancer cells by activating the paracrine effect of the CD44-Rac1 signaling pathway and it is a unique SLRP that promotes the progression of scirrhous gastric cancer and is required for the cooperative invasion of cancer-associated fibroblasts and cancer cells.^[39]^ Recently, breast cancer has surpassed lung cancer as the most prevalent form of malignant tumor found in females around the globe.^[38,44]^ Some researchers discovered that ASPN upregulation may play a significant part in the local invasion of breast ductal carcinomas, demonstrating the important role of ASPN in the TGF-β1 promoting EMT process.^[45]^ However, other scientists discovered that ASPN is a fibroblast-derived inhibitor of TGF-β1 and a tumor suppressor associated with good breast cancer prognosis, therefore, high expression of ASPN, which is a stroma-derived inhibitor of TGF-β1, is significantly associated with less aggressive tumors,^[46]^ which reveals the dual role of ASPN in breast cancer.^[47]^ In short, the EMT mechanism, in which ASPN is fully involved, is significant in the occurrence and progression of many cancers. Subsequently, in a variety of cancers, ASPN can either cause or inhibit cancer through EMT.

## 6. Relationship between ASPN and ECM behavior in bone and joint diseases

OA is one of the most frequent form of chronic joint disease.^[48]^ It affects the majority of people over the age of 65 and is the leading cause of mobility limitation in seniors due to musculoskeletal conditions.^[47,49]^ Multiple studies have confirmed and demonstrated that ASPN has many deep-seated connections in the field of bone and joint, particularly OA, possibly through controlling and modifying the TGF-β/ECM system.^[1]^

Liu et al^[50]^ have found that ASPN overexpression promotes OA progression and deterioration, while ASPN inhibition restores chondrocyte homeostasis and delays chondrocyte senescence, resulting in a significant attenuation of medial meniscus induced OA. So ASPN can be inhibited by miR-26b-5p, which is significantly downregulated in OA cartilage and contributes to the exacerbation of experimental OA by inhibiting the TGF-1/Smads pathway in chondrocytes.^[50]^ Kou et al^[51]^ indicate that TGF-β induces ASPN through Smad3 indirectly because TGF-β failed to increase activity through the ASPN promoter. As an important diagnostic gene for OA,^[52]^ ASPN may become a promising target or biomarker for the treatment of OA. DD is a multifaceted chronic process that alters the structure and function of intervertebral discs, and the dysregulation of miRNAs is associated with various pathologies in DD.^[53,54]^ Song^[55]^ showed that individuals with the D14 allele had an increased risk carrying an overall odds ratio of 1.70 (*P* < .05), suggesting that ASPN may be a common risk factor for OA and lumbar-disc degeneration. In addition, other researchers found that ASPN levels were significantly higher in the patient group, especially the male patient group, compared to the control group (*P* < .05), so ASPN appears to be an important biomarker in temporomandibular joint disease.^[56]^ In the process of intervertebral DD, some of researchers discovered that IL-1 increases ASPN expression via the NF-B p65 pathway in nucleus pulposus cells, whereas IL-1 increases ASPN expression via the same pathway.^[57]^ ASPN was first discovered in bone and cartilage and its research is the most extensive and in-depth.^[2,3]^ The above studies have shown that ASPN can affect TGF-β/Smads pathway to inhibit the deposition of collagen in bone and joints, and finally, cause OA and joint degeneration.

## 7. Relationship between ASPN and ECM behavior in cardiovascular disease

The most common illnesses seen aging society are cardiovascular disorders.^[58]^ In point of fact, becoming older is a factor in the structural and functional deterioration of both the heart and the blood circulation system.^[59]^ The EMT mechanism is critical in cardiac remodeling, myocardial fibrosis, and other pathologies that occur in HF, DCM, and myocardial infarction.^[60,61]^ ASPN can be activated by EMT mechanisms induced by TGF-β to promote cardiac fibrosis.^[62,63]^

Li et al^[62]^ found that upregulation of ASPN was important in cardiomyocyte apoptosis and hypertrophy induced by glycated low-density lipoproteins (gly-LDL) while Downregulation of ASPN prevented gly-LDL induced cell apoptosis and fibrosis. Moreover, any of the EMT-related signaling pathways have been shown to be required for proper embryonic heart development, and cardiac can be repaired by epicardial EMT.^[13]^ Liu et al^[64]^ findings suggest that LINC00636, which is found in the human pericardial fluid, is an antifibrotic molecule that inhibits MAPK1 via miR-450a-2-3p overexpression and improves cardiac fibrosis in atrial fibrillation patients. Zhank et al^[32]^ obtained 59 common genes from differentially expressed genes associated with ischemic cardiomyopathy and DCM, which are mainly involved in cardiac fibrosis and multiple signaling pathways. There are 6 hub genes with the highest degree, among which ASPN has a higher correlation with left ventricular ejection fraction.^[32]^ Zhang et al^[33]^ identify gene connections, interaction networks, and molecular regulatory mechanisms using weighted gene coexpression network analysis, crosstalk analysis, and Pivot analysis in HF. ASPN is 1 of 5 up-regulated hub genes identified in HF, which may provide new insight into the mechanisms underlying HF and aid in the identification of more effective HF therapeutic targets.^[33]^ Similarly, another GEO bioinformatics analysis believes that ASPN is one of the hub genes of ischemic cardiomyopathy, while the expression of ASPN is found to be contrary to the GEO analysis results when qPCR verification is performed on heart tissue from heart transplant patients.^[65]^ There was also evidence that patients undergoing tetralogy of fallot repair had higher mRNA expression of genes associated with connective tissue diseases and remodeling, such as the ASPN, even if their protein levels were not significantly higher in these individuals.^[66]^ In short, it can be seen from the above analysis that ASPN, as a matrix-related substance, participates in the process of epithelial-mesenchymal transformation in the field of cardiovascular disease through a variety of pathways.

## 8. Relationship between ASPN and ECM Behavior in keloid disorder

Keloid disorder is a group of fibroproliferative skin diseases, which is in response to local mechanical stimuli, characterized by unrelenting accumulation of ECM, primarily collagen, resulting in cutaneous tumors on predilection sites of skin mainly by TGF-β signaling.^[67–69]^ Moreover, keloids, in contrast to other fibrotic diseases and normal wound healing, show the little transformation of dermal fibroblasts into α-SMA^ + ^myofibroblasts.^[35]^

Experiments with human dermal cells showed that ASPN is the most strongly expressed gene in keloids and its gene-ontology terms relate strongly to ECM, and dermal injection of ASPN-overexpressing human dermal cells into murine wounds recapitulated keloid collagen histopathological characteristics.^[35]^ Therefore, they draw a conclusion that ASPN inhibits collagen matrix-mediated intercellular mechanocommunications between fibroblasts during keloid progression.^[35]^ But as an antifibrotic protein, ASPN, was found to be a differentially expressed protein identified in normal skin and keloid scar in another study, which indicates that the role of ASPN in the development of keloid may not be as simple as previously thought.^[34]^ Arjunan et al^[70]^ found that human Wharton jelly stem cell-conditioned medium (hWJSC-CM) inhibited the growth of Asian keloid cells both in vitro and in xenograft mice, suggesting that it could be used as a novel treatment option for keloids in humans. But Culley et al^[71]^ found that ASPN expression was lower in c64a fibroblasts compared to 3 in-house fibroblast lines, regardless of TGF-1 treatment, which is subject to further study. Finally, we can conclude that ASPN is very complex in keloids, but as research progresses, ASPN has the potential to be a promising biomarker and therapeutic target for keloid formation.

## 9. Relationship between ASPN and ECM behavior in PF

PF is a chronic, progressive, fibrotic, and heterogeneous disorder of lung interstitial tissue.^[72]^ There are many different causes of PF, connective tissue disease-associated interstitial lung disease has a distinctive histopathologic pattern and may be treated with immunosuppressive drugs, such as glucocorticoids and cyclophosphamide,^[73,74]^ while idiopathic PF is a fatal fibrotic lung disease that is resistant to immune system-targeting drugs and often has a poor prognosis, which is treated by antifibrotic drugs.^[75–77]^ ASPN is also one of the hub genes of PF and is involved in the EMT process of PF.^[78–80]^

According to the results of a recent study,^[36]^ PF mouse models had higher levels of ASPN expression, and ASPN was primarily found in α-SMA^ + ^myofibroblasts. Furthermore, ASPN knockdown inhibited TGF-β/Smads signaling by suppressing the recycling of TβRI to the cell surface in a Rab11-dependent manner and facilitating lysosome-mediated degradation of TβRI, according to additional molecular mechanisms.^[36]^

## 10. Conclusion

Recent studies have given insight into the regulation and function of the protein known as ASPN, which serves as an initial introduction to the members of the SLRPs family. Even though the control of ASPN has been clearly shown in many disease models employing human and mouse tissue samples and cells, the role of ASPN in EMT remains poorly understood. Briefly, collagen deposition and EMT are 2 of the most important pathological processes that are implicated in the pathogenesis of a wide variety of diseases, including cancer, bone and joint diseases, cardiovascular disease, keloid condition, PF, and others, and ASPN can interact with collagen-related proteins such as CAL3A1 and CAL1A1 via TGF-β, Wnt/β-catenin, Notch, and other pathways, hence participating in the EMT mechanism. To date, ASPN’s protein translation process, the co-expression network of ASPN with other genes, and the regulation mechanism of its upstream noncoding RNAs are not well characterized. Given the significance of ASPN in collagen behavior, tailored therapy for disorders associated with collagen deposition will be particularly promising in the future. The research on ASPN might make it possible to come up with innovative approaches to treatment for medical conditions.

## Author contributions

**Data analysis:** Rui Fan.

**Data collection:** Rui Fan.

**Study design:** Xiao-yan Yan, Rui Fan.

**Writing—original draft:** Rui Fan.

**Writing—review and editing:** Wei Zhang.
